# Ultrafast structural dynamics of photo-reactions observed by time-resolved x-ray cross-correlation analysis

**DOI:** 10.1063/1.5086374

**Published:** 2019-03-13

**Authors:** Peter Vester, Ivan A. Zaluzhnyy, Ruslan P. Kurta, Klaus B. Møller, Elisa Biasin, Kristoffer Haldrup, Martin Meedom Nielsen, Ivan A. Vartanyants

**Affiliations:** 1 Department of Physics, Technical University of Denmark, DK-2800 Lyngby, Denmark; 2 Deutsches Elektronen-Synchrotron DESY, Notkestraße 85, D-22607 Hamburg, Germany; 3 National Research Nuclear University MEPhI (Moscow Engineering Physics Institute), Kashirskoe shosse 31, 115409 Moscow, Russia; 4 European XFEL, Holzkoppel 4, D-22869 Schenefeld, Germany; 5 Department of Chemistry, Technical University of Denmark, DK-2800 Lyngby, Denmark; 6 PULSE Institute, SLAC National Accelerator Laboratory, Menlo Park, California 94025, USA

## Abstract

We applied angular X-ray Cross-Correlation analysis (XCCA) to scattering images from a femtosecond resolution X-ray free-electron laser pump-probe experiment with solvated PtPOP {[Pt_2_(P_2_O_5_H_2_)_4_]^4–^} metal complex molecules. The molecules were pumped with linear polarized laser pulses creating an excited state population with a preferred orientational (alignment) direction. Two time scales of 1.9 ± 1.5 ps and 46 ± 10 ps were revealed by angular XCCA associated with structural changes and rotational dephasing of the solvent molecules, respectively. These results illustrate the potential of XCCA to reveal hidden structural information in the analysis of time-resolved x-ray scattering data from molecules in solution.

## I. INTRODUCTION

Recent development of coherent X-ray sources, such as synchrotrons and X-ray free-electron lasers (XFELs), has led to substantial progress in time-resolved X-ray scattering techniques, which allows one to study structural dynamics on the femtosecond scale, making it possible to track chemical reactions in real time.^1^ Despite significant progress of X-ray scattering methods over the last few decades, investigations of the molecular structure and dynamics remain a challenging experimental task. A general problem within the structural analysis framework of small- and wide-angle X-ray scattering (SAXS/WAXS) experiments from molecules in solution is to deduce a large number of structural parameters, including the three-dimensional (3D) structural model of the molecules and their interactions with the surrounding solvent molecules, from a single azimuthally integrated one-dimensional (1D) scattering curve. Moreover, the key structural parameters deduced from conventional SAXS/WAXS experiments are known to be strongly correlated with experimental parameters such as the excitation fraction,^2,3^ which further complicates the evaluation of the molecular structure from the experimental data.

A possible way to enhance the structural information obtained in X-ray experiments is to excite molecules by a polarized pump laser which may introduce orientational anisotropy in the distribution of bond lengths and angles in the photoexcited solute molecules^4^ and utilize anisotropic information recorded by two-dimensional (2D) X-ray detectors for better optimization of the structural models. In this respect, angular X-ray cross-correlation analysis (XCCA)^5–11^ has significant potential to extract and utilize anisotropic information contained in 2D diffraction patterns to provide additional constraints for structural models in the framework of conventional SAXS/WAXS analysis and, importantly, reveal otherwise hidden information on the structure and dynamics of molecules under investigation.

The method of angular intensity correlations in X-ray diffraction goes back to a pioneering work of Kam^12^ and has been recently further developed in a number of publications (for a review, see Ref. 13). This method can, in principle, reveal information about the structure of an individual particle in solution, not available from azimuthally integrated SAXS/WAXS measurements. Moreover, the angular distribution of scattered X-ray intensity also contains information about the spatial orientation of molecules. The ultra-bright femtosecond X-ray pulses from XFELs provide an opportunity for measuring scattering signals with a time resolution much shorter than rotational relaxation times, enabling studies of molecular rotational dynamics by laser pump/X-ray probe experiments. In this case, XCCA offers a model independent approach for investigation of structural dynamics of photo-excited ensembles of particles (molecules, proteins, etc.) in solution. In contrast to conventional SAXS/WAXS techniques, XCCA automatically separates scattering of bulk (isotropic) solvent from anisotropic solute in the experimental scattering data. Our studies show that XCCA may significantly enhance the information content of scattering images from systems of partially oriented solvated molecules. The present work is a novel application of XCCA to investigate the structure and dynamics of solvated molecules.

This work is focused on the analysis of experimental data obtained in the laser pump/X-ray probe scattering experiment performed at the Linear Coherent Light Source (LCLS).^14,15^ In contrast to these publications where the interpretation was based on a theoretical model of the molecular structure in solution, XCCA allowed us to study the molecular dynamics without any *a priori* knowledge or assumptions (cosine squared distribution of photo-excited molecules, symmetric top shape of the molecules, etc.). As such, this work demonstrates for the first time the validity of the XCCA technique to elucidate the structure and dynamics at the molecular level. To verify our findings, we compare experimental results with simulated X-ray diffraction patterns obtained from density functional theory (DFT) calculations of the molecular structure.

## II. EXPERIMENT

**FIG. 1.**
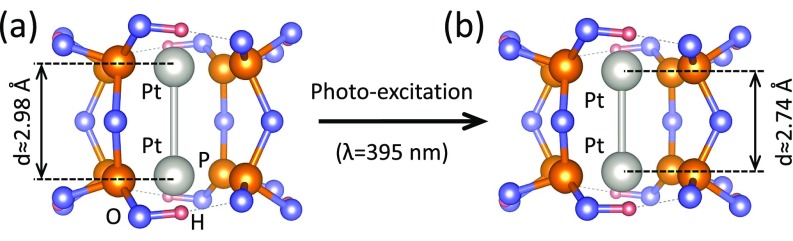
(a) Structure of the PtPOP molecule in the ground state. (b) Structure of the PtPOP molecule in the excited state. Contraction of about 0.24 Å between two Pt atoms is shown.

**FIG. 2.**
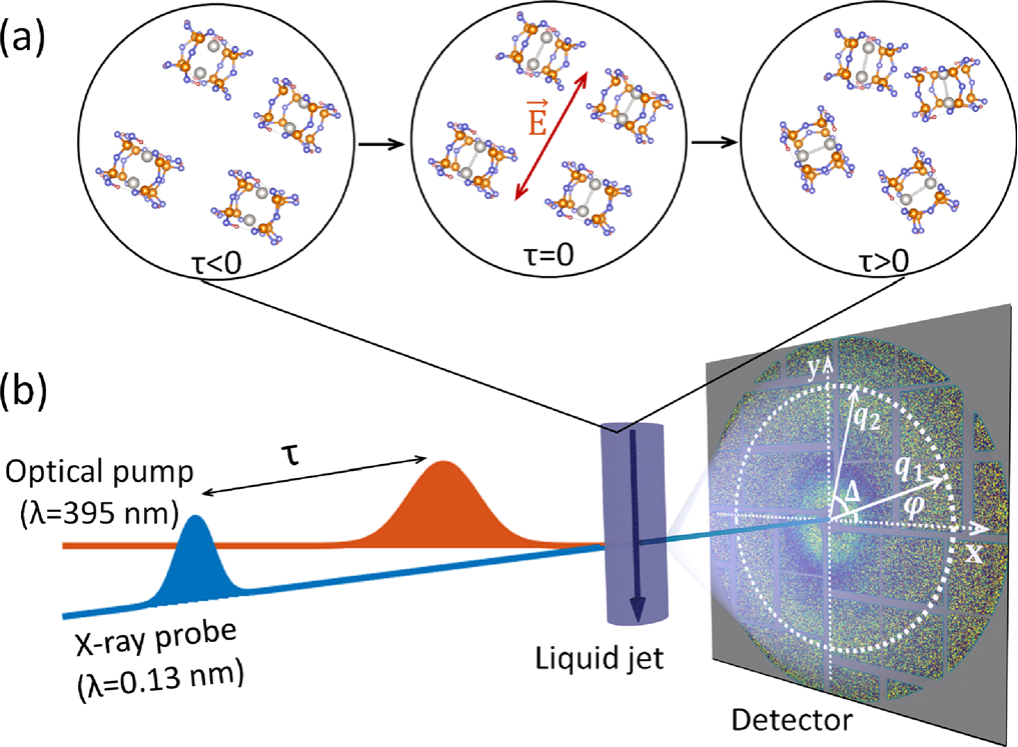
(a) Temporal evolution of an ensemble of randomly oriented PtPOP molecules before (*τ* < 0) and after (*τ* ≥ 0) excitation. The optical pump selectively excites PtPOP molecules with the transition dipole moment (along the Pt-Pt axis) parallel to the laser electric field E of the excitation laser at *τ* = 0, and the population of excited molecules eventually evolves (*τ* > 0) to a random orientational distribution on a timescale of tens of picoseconds. (b) Scheme of the pump-probe experiment at LCLS. The experiment utilizes the optical pump laser/X-ray probe detection scheme on a circular liquid jet system with the time resolution given by the time delay *τ* of the femtosecond X-ray pulse. On the detector, momentum transfer vectors *q*_1_ and *q*_2_ are shown with the angular coordinates *φ* and *φ* + Δ.

The investigated metal complex molecules tetrakis-*μ*-pyrophosphitodiplatinate(II) anion {[Pt_2_(P_2_O_5_H_2_)_4_]^4–^, PtPOP} consist of two square-planar Pt units held together by four pyrophosphito ligands [Fig. 1(a)]. It belongs to a family of binuclear *d*^8^–*d*^8^ transition metal complexes exhibiting photophysical properties of both fundamental and applied interest^16^ and shows intense luminescence with a total quantum efficiency very close to unity. It is now very well established that upon photo-excitation at or near the 370 nm absorption peak, an electron is promoted from an anti-bonding 5dσ* highest occupied molecular orbital (HOMO) to the bonding 6*pσ* lowest unoccupied molecular orbital (LUMO). This chemical transition leads to a pronounced structural change in the form of contraction^17,18^ of the Pt atoms along the Pt-Pt axis by 0.24(4) Å [Fig. 1(b)]. Furthermore, as the transition dipole moment lies along the Pt-Pt axis, the molecules will be selectively photoexcited as a function of the Pt-Pt axis orientation relative to the polarization of the optical pump pulse,^4^ all of which makes it an excellent candidate for exploring the potential of XCCA (Fig. 2).

Following the photo-excitation event, the molecule is in a singlet state and on a time scale of 1–10 ps undergoes inter-system crossing (ISC) to a triplet state. The excited singlet and triplet states are well separated, both in their respective lifetimes (1–10 ps and 10 *μ*s, respectively) and in terms of their potential energy surfaces, which are highly harmonic, nested potentials shifted 0.24(4) Å along the Pt-Pt coordinate also with respect to the highly harmonic ground state potential surface.^16,19^

The dynamics following photo-excitation of aqueous PtPOP molecules was tracked in time-resolved pump-probe X-ray diffuse scattering (XDS) experiments at the X-ray Pump-Probe (XPP) beamline of the LCLS XFEL facility as schematically illustrated in Fig. 2 (for details of the experimental setup, see Refs. 20 and 21). The aqueous solution of PtPOP was excited by a short 50 fs laser pulse with a wavelength of 395 nm (pump) followed by a 50 fs 9.5 keV X-ray pulse (probe) at a certain time delay. The X-ray probe pulses were polarized in the horizontal direction, whereas the laser pump pulses had a linear polarization of 20° off the vertical. Thus, a single pump-probe event gives a snapshot of the configuration after photo-excitation at a single time-delay *τ*, and by combining snapshots at different time-delays, the dynamics of the excited molecules can be followed (see Ref. 14 for the details of experiment).

**FIG. 3.**
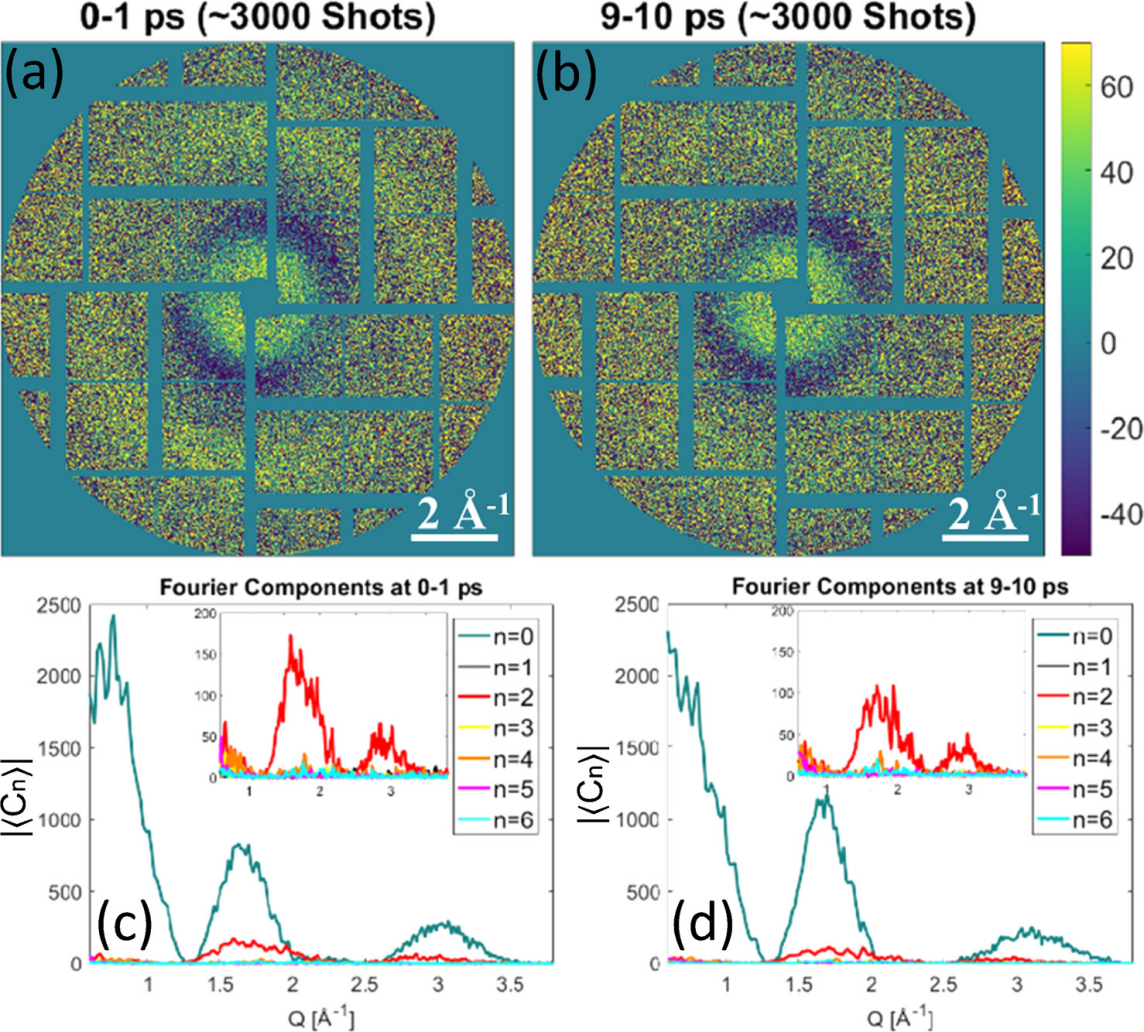
(a) and (b) Difference scattering detector images (laser on – laser off) for two different one picosecond time delay intervals (scattering data are taken from Ref. 14). For better visualisation, diffraction patterns in (a) and (b) were averaged over about 3000 pulses within each time delay interval. (c) and (d) Calculated averaged Fourier components of the CCFs. The insets show the dominant anisotropic *n *=* *2 Fourier component contribution (the isotropic *n *=* *0 Fourier component is removed from the insets).

XDS signals were recorded in the forward direction using a large-area 2D Cornell-SLAC pixel array detector^22^ (CS-PAD) positioned 10 cm behind the sample and corrected for effects such as polarization, solid angle, absorption, background/dark image subtraction, and outlier rejection as previously described.^21^ The diffraction patterns were masked prior to further analysis to exclude the beamstop area, gaps between detector tiles, as well as non-responsive pixels on the detector. The pixels were also binned by 4 × 4 pixel groups to increase the signal-to-noise ratio. In the following, we analysed the difference signal images which are created by subtracting a laser-off (all molecules in the ground state) image from the nearest laser-on images (some molecules in the excited state) in the sequence of collected detector images. The difference scattering images contain only a change in the diffraction signal from the structural differences between ground- and excited-state PtPOP molecules and their interaction with the solvent, while the constant background arising from solvent scattering cancels out. At LCLS, we collected a sufficient amount of diffraction patterns (*M *≈* *3000) within each time bin of 1 ps for reliable XCCA analysis. The measured difference scattering images averaged over 1 ps intervals of two different time delays *τ* = 0–1 ps and *τ* = 9–10 ps are shown in Figs. 3(a) and 3(b), respectively. One can see slight anisotropy in the intensity of the difference scattering images, parallel to the laser polarization (about 20° to the vertical direction), which appears due to the photo-excitation selectively occurring in molecules with the Pt-Pt axis oriented along the polarization vector of the pump laser pulse.^4^

## III. X-RAY CROSS-CORRELATION ANALYSIS

Angular anisotropy of difference diffraction patterns was analyzed by XCCA using two-point angular cross-correlation functions (CCFs). In this work, we apply the CCF defined on the scattering ring of radius *q*, where **q** = (*q*, *φ*) is the momentum transfer vector defined in the polar coordinate system of the 2D detector^13,23^

$$C(q,\Delta )={\left\langle I^{\text{dif}}(q,\varphi )I^{\text{dif}}(q,\varphi +\Delta )\right\rangle }_{\varphi },$$ (1)

where $I^{\text{dif}}(q,\varphi )=I^{\text{on}}(q,\varphi )-I^{\text{off}}(q,\varphi )$ is the measured difference intensity between the laser-on *I*^on^(*q*, *φ*) and laser-off *I*^off^(*q*, *φ*) diffraction patterns, Δ is the angular coordinate, and ${\left\langle f(\varphi )\right\rangle }_{\varphi }$ denotes the angular average of the function *f*(*φ*).

It is convenient to decompose the CCFs using an angular Fourier series on a ring of radius *q*,

$$C(q,\Delta )=\sum_{n=-\infty }^{\infty }C_{n}(q)e^{\mathrm{in}\Delta },$$ (2)

$$C_{n}(q)=\frac{1}{2\pi }\int_{0}^{2\pi }C(q,\Delta )e^{-\mathrm{in}\Delta }d\Delta ,$$ (3)

where *C_n_*(*q*) are the angular Fourier components (FCs) of the CCF. It can be shown^23^ that FCs of the CCFs are directly related to FCs of the difference intensities as

$$C_{n}(q)={\left|I_{n}^{\text{dif}}(q)\right|}^{2}.$$ (4)

In practical applications, one employs CCFs ${\left\langle C(q,\Delta )\right\rangle }_{M}$ and FCs ${\left\langle C_{n}(q)\right\rangle }_{M}$ averaged over a large number *M* (typically of the order of 10^3^ to 10^4^) of diffraction patterns to access ensemble-averaged quantities and improve the signal-to-noise ratio. The ensemble-averaged CCFs and their FCs can be directly related to the structure of molecules and their orientational distribution.^23,24^

It is important to highlight a few key differences of the XCCA approach outlined by Eqs. (1)–(4) for analysis of the time-resolved pump-probe solution scattering data, as compared to established SAXS/WAXS or XDS approaches for analysis of time-resolved experiments.^2,4,14,25^ First, XCCA is based on the analysis of the CCFs rather than scattering intensity, thus enabling direct access to angular high-order scattering contributions associated with FCs ${\left\langle C_{n}(q)\right\rangle }_{M}$ of high orders *n *>* *0, which are unavoidably lost in conventional SAXS/WAXS analysis. The key aspect here is that the averaging of CCFs preserves additional angular structural information hidden in fluctuations of anisotropic intensity distribution, while averaging of intensities in SAXS/WAXS/XDS analyses may, generally, lead to information loss.^13^

Second, as can be seen from Eqs. (1)–(4), XCCA is a model-independent approach since it does not rely on any *a priori* assumptions on the molecular structure or distribution of molecular orientations. The experimentally determined Fourier spectra, specifically the number of non-vanishing FCs ${\left\langle C_{n}(q)\right\rangle }_{M}$ and their orders *n*, as well as their *q* and time dependence, can be used *a posteriori* to test against a model of the system under study. This is in contrast to commonly used approaches for analysis of pump-probe solution scattering data, where the interpretation of anisotropic scattering relies on assumptions regarding a particular model of orientational distribution (e.g., cosine squared) of the excited-state molecules. Consequently, without being constrained by *a priori* model assumptions, the XCCA approach may provide new insights into the dynamics of orientational distributions of molecules, as well as reveal new excitation/relaxation pathways in the system.

Finally, XCCA employs Fourier analysis of the CCFs, a procedure which has inherent separation of isotropic and anisotropic contributions to the measured difference scattering signals, as these contribute to FCs of different orders *n*. Hence, a computationally efficient fast Fourier transform (FFT) algorithm can be used for accurate quantitative analysis of contributions of different orders, without a need to perform any fitting as compared to previous work.^14,26^

## IV. RESULTS AND DISCUSSION

### A. Analysis of the angular anisotropy

XCCA provides an efficient tool for model-independent analysis of angular anisotropy of difference diffraction patterns shown in Figs. 3(a) and 3(b). The averaged Fourier components of the angular CCF (4) from the experimental difference scattering intensities at two different time delays evaluated according to definition (3) are shown in Figs. 3(c) and 3(d). According to Eqs. (1)–(3), the zero-order angular Fourier component (*n *=* *0) can be considered as the square of an azimuthally integrated difference intensity *I*^dif^ (*q*, *φ*),

$$\langle C_{0}(q)\rangle \propto {\left|\int I^{\text{dif}}(q,\varphi )d\varphi \right|}^{2}.$$ (5)

The first two peaks of $\left\langle C_{0}(q)\right\rangle$ at *q *≈* *0.7 Å^−1^ and *q *≈* *1.8 Å^−1^ [see Figs. 3(c) and 3(d)] correspond to internal concentric rings with positive and negative signals on the difference scattering images in Figs. 3(a) and 3(b). A clearly visible peak of the same Fourier component at *q *≈* *3.0 Å^−1^ [see Figs. 3(c) and 3(d)] corresponds to the broad anisotropic external scattering ring in Figs. 3(a) and 3(b).

Information about angular anisotropy in diffraction can be conveniently accessed by evaluation of the higher-order Fourier components of the CCF $\left\langle C_{n}(q)\right\rangle$, i.e., for *n *=* *2, 4, 6,…. A dominant *n *=* *2 angular Fourier component in the diffraction pattern is clearly visible in the inset of Figs. 3(c) and 3(d). A rapid decay of the dominant component after optical pump pulse is a strong evidence that the observed *n *=* *2 signal arises from a twofold symmetric orientational distribution of the excited state of the molecules induced by the laser excitation at time delay *τ* = 0. Interestingly, weak but consistent *n *=* *4 and *n *=* *6 components can also be seen at around *q *≈* *1.8 Å^−1^. In principle, higher-order intensity Fourier components in the X-ray scattering may originate from the changes in internal symmetry of the individual molecules [see Fig. 1(a)], which was directly observed in simulations with a relatively small number of illuminated particles.^27^ Alternatively, it may indicate deviations from the expected cosine squared distribution of photo-excited molecules or multi-photon excitations of PtPOP molecules. Additional FCs may also indicate structural changes in PtPOP molecules which are not symmetric with respect to the laser polarization axis, e.g., shift of the atoms in the plane perpendicular to the Pt-Pt axis. While it is difficult to make a definite conclusion about the origin of these higher-order FCs in the present case, due to the low signal-to-noise ratio achieved for the available number of diffraction patterns, it is quite promising that model-independent XCCA is capable of revealing additional angular structural contributions.

In general, the azimuthally averaged *n *=* *0 signal contains contributions from the solute, solvent cage, and bulk solvent (heating and density changes caused by the energy transfer from photo-excited solute molecules).^2^ The latter two should be subtracted from the azimuthally average data to extract the signal corresponding to the changes in the solute structure.^28^ In contrast, the *n *=* *2 signal is not influenced by the bulk solvent contributions but includes contributions from variations with the same symmetry as the orientational distribution of the molecules, e.g., specific solvation within the solvent cage. This is, for instance, the case for the bimetallic Ir_2_[dimen]_4_ complex, where acetonitrile molecules were observed to specifically coordinate to the axial Ir sites following photo-excitation^29^ although for PtPOP, such coordination has only been discussed from a theoretical point of view.^19,30^ This significantly reduces the amount of free parameters in any structural analysis, makes the interpretation of the time constants more clear, and requires less assumptions about the system. A combination of both the *n *=* *0 and *n *=* *2 curves will give two different signals to benchmark the structural models, where one is dependent and the other is independent of the bulk solvent contribution, as recently demonstrated.^15^

### B. Analysis of molecular dynamics

**FIG. 4.**
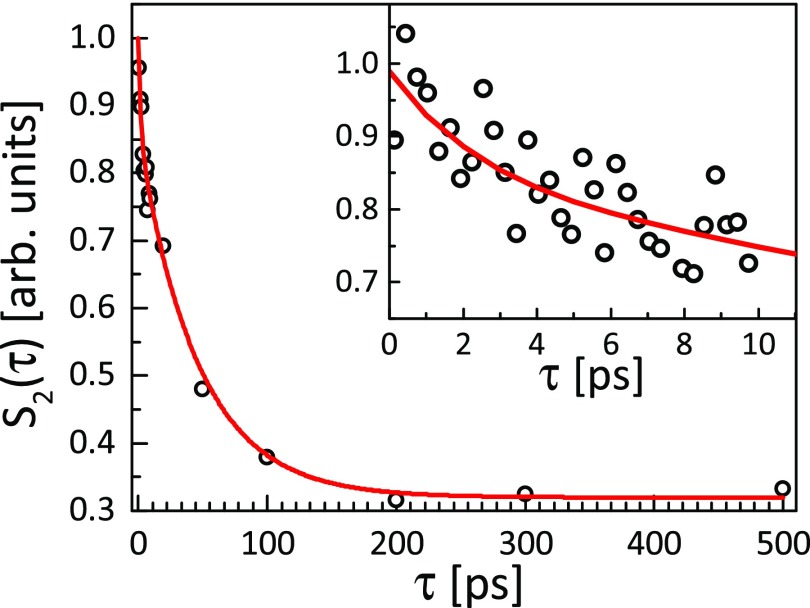
A double exponential decay with the time constants *τ*_1_ = 1.9 ± 1.5 ps and *τ*_2_ = 46 ± 10 of the normalized area $S_{2}(\tau )=\int \sqrt{\left\langle C_{2}(q,\tau )\right\rangle }dq$ under the peak at *q *=* *1.8 Å^−1^. The average Fourier components of CCF $\left\langle C_{2}(q,\tau )\right\rangle$ were calculated according to Eq. (4) binned in 1 ps bins. In the inset, binning into 250 fs time bins reveals short time decay. Points are experimental data, and the red solid line is a fit made by Eq. (6).

The values of non-zero CCF Fourier components contain all available information about the orientational distribution of photo-excited molecules. To study the dynamics of excited PtPOP, we considered the temporal evolution of the *n *=* *2 Fourier component. To quantify the fraction of excited molecules within the cosine squared orientational distribution, the normalized integrated area $S_{2}(\tau )=\int \sqrt{\left\langle C_{2}(q,\tau )\right\rangle }dq$ under the peak at *q *≈* *1.8 Å^−1^ of the averaged second-order CCF Fourier component was considered (integration over *q* was performed within the range of 1.1 Å^−1^–2.3 Å^−1^). The temporal evolution of this quantity as a function of delay time *τ* = 0–500 ps is shown by dots in Fig. 4. One can clearly see a significant decay of the anisotropic signal in the selected time range. Binning of the same data into 250 fs time bins, shown in the inset of Fig. 4, reveals an additional short-time decay, which is clearly visible on top of the longer decay. Thus, the total decay was approximated by a sum of two exponential terms

$$S_{2}(\tau )=Ae^{-\tau /\tau_{1}}+Be^{-\tau /\tau_{2}}+C,$$ (6)

where *A *=* *21 ± 5% and *B *=* *79 ± 5% are scale constants and the constant *C* arises from the pixel-to-pixel noise of the detector images (see Fig. 3) and as such represents the noise level (a signal-to-noise ratio of approximately 3 at short times) of the data. Based on least-squares fitting (see the solid line in Fig. 4), the values of time constants were found to be *τ*_1_ = 1.9 ± 1.5 ps and *τ*_2_ = 46 ± 10 ps. Including the second exponential term in the fitting sufficiently decreased (by almost 20%) the value of the reduced *χ*^2^. We would like to stress here that the values of time constants were obtained without any modeling or *a priori* knowledge of system behavior, as compared to previous work,^14^ where a cosine squared distribution of excited state molecules was assumed from the beginning of the analysis.

The longer time scale *τ*_2_ = 46 ± 10 ps may be interpreted as the rotational dephasing of the initial cosine squared distribution of orientations to a completely random and isotropic distribution. The reorientation time *τ_r_* for a molecule in solution can be estimated from the Stokes-Einstein-Debye hydrodynamic theory in a classical dynamical framework without electrical interactions as *τ_r_* ≈ 50 ps.^14^ This estimate is in good agreement with both the present result (*τ* = 46 ± 10 ps) as well as with the result obtained by evaluating the ratio between Δ*S*_0_ and Δ*S*_2_ components in Ref. 14 (*τ* = 60 ± 10 ps) and we note that the confidence intervals on the two experimental estimates overlap, despite the significant differences in analysis applied to obtain these results. The specific molecular shape of PtPOP can be taken into account (here, we approximated the shape of the PtPOP molecule by a sphere with the radius *r *=* *4 Å), which would lead to a slight change in the value of rotational time constant *τ_r_*. It means that the *n *=* *2 contribution from the ground and excited states can be distinguished by following the temporal evolution of the *n *=* *2 signal as the molecules with a longer time constant will dominate the signal at longer time delays. This applies as well to experiments where different species (or the same species of different sizes) in the same sample volume are excited with a preferred orientational direction. The molecular reorientation time obtained from the *n *=* *2 signal cannot be determined from radially integrated intensity curves and can be used to refine structural models and gain new insight into time-resolved SAXS/WAXS experiments. The shorter time constant *τ*_1_ = 1.9 ± 1.5 ps is in a very good agreement with the dampening time for Pt-Pt vibrations in both the ground- and excited states^15,19,31,32^ and is therefore assigned as arising from vibrational decoherence of the molecule following photo-excitation, which activates vibrational dynamics along this coordinate.

### C. Model of the scattering signal

The time-dynamics results of XCCA can be directly compared with simulations based on a DFT structural model. In this work, we utilize a simple model for the excited state population of PtPOP, where they are considered as linear molecules. Such a model can be justified by rotational symmetry of the PtPOP molecule around the Pt-Pt axis and the fact that the excitation of the molecule can be approximated to a high accuracy by Pt-Pt bond contraction. When such symmetric top molecules are excited from thermal equilibrium by one-photon absorption, the orientational distribution of excited molecules will have a cosine squared distribution with respect to the polarization of the incoming optical photons,^33^ which was indeed observed in the collected X-ray diffraction patterns (Fig. 3).

As a model system for simulation, we assume a 3D disordered sample consisting of *N *=* *10^5^ molecules. In the approximation of a dilute disordered sample where the mean distance between the molecules is larger than the coherence length of the incoming beam, interference between the X-rays scattered from different molecules can be neglected and the total scattered intensity can be represented as a sum of intensities from the individual molecules in the system. The X-ray intensity scattered from one molecule can be evaluated as

$$I_{\text{mol}}(q)={\left|\sum_{i}f_{i}(q)e^{iqr_{i}}\right|}^{2},$$ (7)

where **r**_*i*_ are the atomic positions and *f_i_*(*q*) are the atomic form factors of the i-th atom in the PtPOP molecule.

The coordinates of atoms in excited and ground state PtPOP structures were calculated by DFT simulations (for details, see Refs. 19 and 34) This gives a Pt-Pt bond contraction of 0.24 Å, while the ligand cage structure remains rigid, which is in agreement with experimental X-ray scattering results.^17,18^

To obtain the diffraction patterns from an ensemble of molecules in the excited state, we simulated our sample as one in which each molecule was rotated within the cosine squared distribution. The positions of the atoms in the rotated photo-excited PtPOP molecule were used to calculate a diffuse X-ray scattering signal from a single molecule using Eq. (7). Then, the diffraction signal was averaged over *N *=* *10^5^ molecules, and a difference scattering signal was calculated to obtain similar diffraction patterns as we observed in the XDS experiment.

**FIG. 5.**
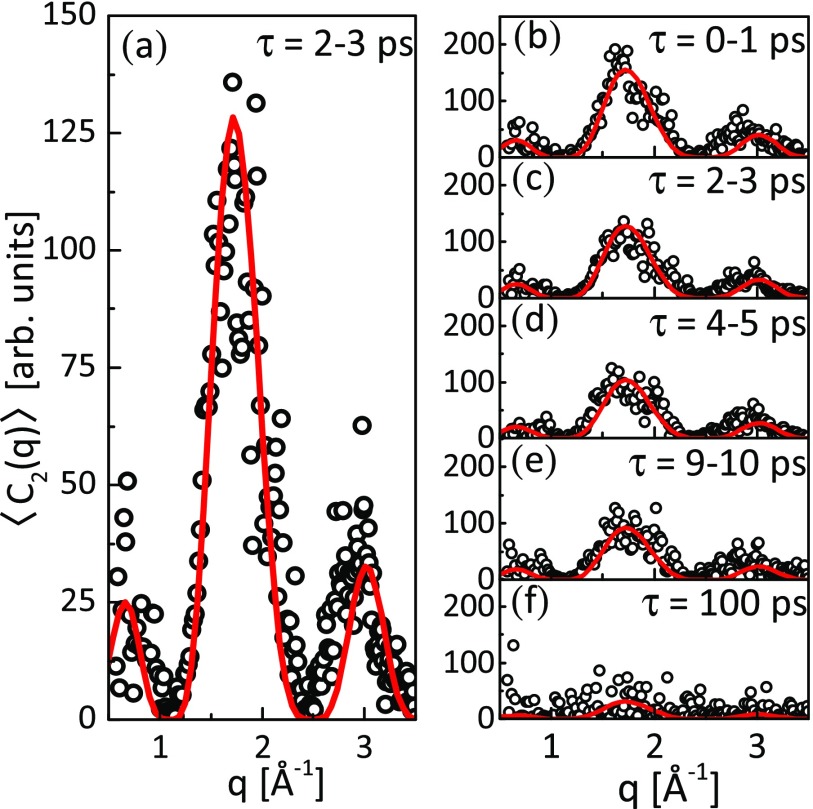
(a) Direct comparison of the calculated *n *=* *2 CCF Fourier components from the simulation of 10^5^ PtPOP molecules with a cosine squared angular distribution, fitted to the measured components in the 2–3 ps time delay interval with a scaling factor *α*(*τ*). (b)–(e) The same comparison for different time delays; (f) at *τ* = 100 ps, the molecules have almost completely lost their preferred orientation. Here, dots are experimental data obtained from XCCA analysis and solid lines are theoretical fits.

Figure 5 shows a comparison of the experimentally observed (dots) and simulated (lines) *n *=* *2 Fourier components of the CCF at different time delays. The source of small discrepancies between the model and experimental data could possibly be related to the H_2_O cage not being included in the DFT modelling. The model signal was scaled with a factor *α*(*τ*) to account for the total number of excited PtPOP molecules in the probed volume of the sample and their orientational distribution at the specific time delay. The scaling factor *α*(*τ*) has a similar temporal decay behavior as observed for the *S*_2_(*τ*) factor shown in Fig. 4, with two time constants of 1.9 ± 1.4 ps and 41 ± 8 ps.

In this work, we assumed that excited singlet and triplet states of the PtPOP molecule are characterized by almost the same Pt-Pt distance within the accuracy of 0.01 Å as indicated by theoretical^19^ and optical studies,^35^ which means that we could not observe singlet-to-triplet ISC in our pump-probe experiment. Therefore, the temporal evolution of the scaling coefficient *α*(*τ*) can be attributed only to the orientational dephasing of the excited molecules and not to the relaxation of the molecules to the ground state since the lifetime of the triplet excited state is known to be about 10 *μ*s. A direct comparison confirms the validity of the assumed model of the system, and the small discrepancies are interpreted as arising from the lack of a simulated solvent cage signal and slight variations between the DFT simulated structure and the actual structure of the molecule. The results of this section demonstrate that *a posteriori* modeling of the XCCA results can be performed to study time-resolved structure dynamics in pump-probe solution experiments.

## V. CONCLUSIONS AND OUTLOOK

In this work, by applying XCCA, we show that the difference signal from photo-excited PtPOP molecules can be well represented by contribution of two (zero- and second-order) Fourier components. The dominant anisotropic signal of the *n *=* *2 FC of the CCF clearly indicates that the orientation of the photo-excited PtPOP molecules can be approximated by a cosine squared distribution. This is in agreement with theoretical predictions,^4,25^ assuming a single-photon excitation and initially non-occupied rotational and vibrational degrees of freedom of the PtPOP molecules.^14^ The results of the XCCA approach presented here are in quantitative agreement with the results of XDS analysis of the same dataset discussed previously in Refs. 14 and 15, indicating the full feasibility of the XCCA approach for analysis of time-resolved pump-probe solution scattering data. Further, the observation of small values of the high-order FCs observed in the present study may indicate the potential for model independent XCCA to go beyond the common assumption of cosine squared distribution of molecular orientations and gain new insight into the structure and dynamics of excited molecules.

Time-dependent analysis of the anisotropic scattering signal reveals that its shape remains unchanged, while the amplitude exponentially decreased to the noise level with two characteristic time-scales *τ*_1_ = 1.9 ± 1.5 ps and *τ*_2_ = 46 ± 10 ps. Taking into account the long lifetime of the photo-excited state of PtPOP molecules and the fact that the ISC from the singlet to the triplet state occurs with near-unity efficiency, this decay can be attributed exclusively to rotational dephasing of molecules (longer time constant) and to internal dynamics of molecules (shorter time constant). Our analysis is supported by simulations which show that the anisotropic scattering can be modelled by the difference scattering signal from excited- and ground-state molecules at any time-delay. In principle, it should be possible to observe oscillations on a sub-300 fs timescale in the difference scattering signal, which is directly related to the Pt-Pt bond stretching mode.^15,19,31^ The direct studies of the bond dynamics would require collecting significantly more scattering patterns with short time delays to accumulate sufficient statistics for the XCCA. This opens up the possibility to investigate, for example, the optical Kerr effect,^36–38^ which is based on the creation of induced dipoles in the solvent molecules by the oscillating light field during the first few hundred femtoseconds after the pump pulse.

In summary, we have shown how XCCA can be applied as a model independent approach to study the structural symmetries and their timescale of a disordered sample of solvated photo-excited PtPOP molecules with a preferred photo-induced orientation. We believe that the technique presented here can be widely used in SAXS/WAXS experiments to enhance structural information from a disordered sample of molecules, proteins, or biomolecules and to reveal hidden symmetries and their time evolution in a model independent approach.
